# Genetic Assessment and Clinical Correlates in Severe Hypertriglyceridemia: A Systematic Review

**DOI:** 10.3390/genes16111377

**Published:** 2025-11-11

**Authors:** Carmine De Luca, Paola Ciciola, Guido D’Errico, Maria Donata Di Taranto, Giuliana Fortunato, Carina Gross, Jonathan Garn, Gabriella Iannuzzo, Matteo Di Minno, Ilenia Calcaterra

**Affiliations:** 1 Department of Clinical Medicine and Surgery, University of Naples Federico II, 80131 Naples, Italy; deluca.carmine.medicina@gmail.com (C.D.L.); paola.ciciola@unina.it (P.C.); guidoderrico1@gmail.com (G.D.); gabriella.iannuzzo@unina.it (G.I.); matteo.diminno@unina.it (M.D.M.); 2 Department of Molecular Medicine and Medical Biotechnologies, CEINGE Advanced Biotechnologies S.C. A R.L, University of Naples Federico II, Via S. Pansini 5, 80131 Naples, Italy; mariadonata.ditaranto@unina.it (M.D.D.T.); giuliana.fortunato@unina.it (G.F.); 3 Faculty of Medicine, Hannover Medical School (MHH), 30625 Hannover, Germany; carina.gross@stud.mh-hannover.de (C.G.); jonathan.garn@stud.mh-hannover.de (J.G.)

**Keywords:** familial chylomicronemia syndrome (FCS), metabolic dysfunction-associated steatotic liver disease (MASLD), polygenic hypertriglyceridemia, precision medicine, severe hypertriglyceridemia

## Abstract

**Background**: Severe hypertriglyceridemia (SHTG) is associated with acute pancreatitis, metabolic dysfunction, and increased cardiovascular risk. Its genetic architecture ranges from rare biallelic variants causing familial chylomicronemia syndrome (FCS) to more prevalent polygenic or multifactorial chylomicronemia syndromes (MCS). **Methods**: We systematically reviewed scientific literature up to 2025 for studies reporting genetic data, clinical features, or therapeutic outcomes in adults with triglycerides (TG) ≥ 500 mg/dL. Extracted data were synthesized for genotype, polygenic risk score (PRS), TG levels, metabolic comorbidities, hepatic steatosis, pancreatitis, and treatment response. **Results**: Ten studies (n = 2521) were included. FCS due to biallelic *LPL*, *APOC2*, *GPIHBP1*, or *LMF1* variants accounted for <5% of cases and showed extreme TG elevations (>2800 mg/dL) with pancreatitis prevalence (>70%). *APOA5*, *APOC3*, and *APOB* variants were associated with intermediate TG levels and high rates of metabolic dysfunction-associated steatotic liver disease (MASLD). Polygenic hypertriglyceridemia represented ~70–80% of cases, with TG ≈ 2200 mg/dL and pancreatitis prevalence 15–20%, largely modulated by metabolic triggers. MASLD was present in >70% of polygenic cases, supporting a “two-hit” model where hepatic overproduction of TG-rich lipoproteins amplifies TG excess. Interventional trials demonstrated TG reductions with APOC3 antisense therapy (70–80%) and ANGPTL3 inhibition (50–55%), while GLP-1RA significantly reduced hepatic fat (30–35%) and resolved NASH in up to 59% of patients. **Conclusions**: SHTG displays a genotype–phenotype gradient: FCS is linked to recurrent pancreatitis, whereas polygenic/MCS forms are closely associated with MASLD and metabolic dysfunction. These findings support a precision-medicine approach integrating genetic testing and PRS-guided strategies—prioritizing APOC3/ANGPTL3 inhibitors for FCS and combined TG-lowering plus metabolic therapies for MCS—to reduce pancreatitis recurrence and liver disease.

## 1. Introduction

Hypertriglyceridemia (HTG) is a subset of dyslipidaemia characterized by elevated plasma triglycerides (TG) levels [1,2], which develops due to hereditary disorders (familial chylomicronaemia syndrome, familial combined hyperlipidaemia, and familial HTG) and several pathologic settings (diabetes, metabolic syndrome, insulin resistance, thyroid disorders) [3,4].

Plasma TG concentration is a biomarker of circulating TG-rich-lipoproteins (TRL) (chylomicrons and very low-density lipoprotein, VLDL) and their metabolic remnants [5]. TRL are known to have a pathogenic role in the development of acute pancreatitis (chylomicrons) [6,7] and of cardiovascular (CV) disease (VLDL and remnants) [8,9].

Severe hypertriglyceridemia (SHTG) is defined as fasting TG ≥ 500 mg/dL; however, TG levels ≥ 1000 mg/dL markedly increase the risk of acute pancreatitis. Although available lipid-lowering therapies (fibrates, statins, and omega-3 fatty acids) decrease triglyceride levels [10,11], a not-negligible number of SHTG subjects do not achieve therapeutic goals [12]. In this regard, SHTG therapeutic management still represents a major unmet clinical need.

SHTG crosses a spectrum ranging from rare monogenic diseases, such as familial chylomicronemia syndrome (FCS), to more frequent polygenic and multifactorial phenotypes influenced by metabolic dysfunction. At one end of the spectrum, biallelic pathogenic variants in *LPL*, *APOC2*, *APOA5*, *GPIHBP1*, or *LMF1* lead to a complete loss of lipolytic function, resulting in early-onset familial chylomicronaemia and fasting triglyceride concentrations typically exceeding 10 mmol/L (≈885 mg/dL). These patients display a classical FCS phenotype characterized by persistent chylomicronaemia, eruptive xanthomas, lipemia retinalis, and recurrent pancreatitis, often refractory to conventional lipid-lowering therapies. On the opposite hand, a polygenic background interacts with metabolic risk factors including obesity, insulin resistance, diabetes, alcohol consumption, and secondary medications to define multifactorial chylomicronemia syndrome (MCS) [3,13]. Between these extremes lies a broad intermediate group carrying heterozygous or low-penetrance variants that modulate disease expression through interaction with environmental and metabolic triggers. Significantly, a phenotypic overlap between FCS and MCS is increasingly recognized, with some patients exhibiting intermediate biochemical and clinical features, such as partial responsiveness to therapy or fluctuating triglyceride levels, reflecting the continuum of genetic and metabolic determinants underlying SHTG. Pragmatic diagnostic algorithms, such as the FCS clinical score (cutoff ≥ 10; sensitivity 88%; specificity 85%), have been proposed to bridge genotype and phenotype, improving early recognition and guiding therapeutic stratification [3].

This progressive model highlights that severe hypertriglyceridemia rarely results from isolated defects in the canonical lipolytic pathway alone, but rather from a broader network of genetic modifiers that influence triglyceride turnover and hepatic lipid flux.

Beyond canonical FCS-related genes, other loci critically influence triglyceride metabolism and hepatic fat accumulation, linking SHTG to systemic metabolic dysfunction [14]. Among these, apolipoprotein C-III (ApoCIII) and apolipoprotein B (ApoB) act as pivotal regulators of triglyceride-rich lipoprotein (TRL) metabolism, bridging lipolytic pathways with hepatic lipid handling and cardiovascular risk [15].

ApoCIII is a 79–amino acid glycoprotein primarily synthesized in the liver and, to a lesser extent, in the intestines. In normolipidemic individuals, it circulates mainly in HDL particles, whereas in hypertriglyceridemic states it predominantly associates with TRLs [16]. This redistribution suggests that ApoCIII may mediate triglyceride exchange between HDL and TRLs through mechanisms that remain incompletely understood. Functionally, ApoCIII inhibits lipoprotein lipase (LPL) activity and impairs the hepatic clearance of TRL remnants via blockade of lipoprotein receptors, underscoring its role in multiple steps of triglyceride metabolism [17].

Mendelian randomization studies have shown that loss-of-function mutations in the APOC3 gene are associated with lifelong low triglyceride levels and reduced cardiovascular risk, whereas overexpression or gain-of-function variants contribute to SHTG [18,19]. Epidemiologic data further demonstrate that elevated ApoCIII levels correlate with increased cardiovascular risk, particularly among patients with diabetes, highlighting its dual role as both a metabolic and vascular risk factor. [20,21]. Inclusion of these genes in extended diagnostic panels enhances etiologic resolution and guides access to emerging therapies.

ApoB, the essential structural component of very low-density lipoproteins (VLDL), intermediate-density lipoproteins (IDL), and low-density lipoproteins (LDL), plays a central role in hepatic lipoprotein assembly and secretion. Each atherogenic particle contains a single ApoB molecule, making ApoB a quantitative index of circulating atherogenic burden. In the context of severe hypertriglyceridemia, partial loss-of-function *APOB* variants can paradoxically increase hepatic triglyceride accumulation by impairing VLDL export, thereby promoting steatosis despite lower plasma cholesterol concentrations [22]. Conversely, hypersecretion of ApoB-containing VLDL particles, often driven by insulin resistance and hepatic overproduction, is a hallmark of polygenic or metabolic hypertriglyceridemia [23]. Together, these findings place ApoB at the intersection between hepatic lipid trafficking and systemic triglyceride excess, linking polygenic dyslipidemia to metabolic dysfunction-associated fatty liver disease (MASLD) and cardiometabolic risk. Incorporating *APOB* into extended diagnostic panels enhances the precision of genetic assessment and provides a mechanistic rationale for emerging therapeutic approaches aimed at modulating VLDL production and secretion, in synergy with agents targeting APOC3 and ANGPTL3.

Importantly, SHTG often coexists with metabolic dysfunction-associated steatotic liver disease (MASLD) [24,25,26], reflecting a bidirectional relationship between hepatic insulin resistance, steatosis, and impaired clearance of triglyceride-rich lipoproteins. This overlap amplifies systemic risk and identifies a subgroup of patients with refractory hypertriglyceridemia (rHTG) who remain uncontrolled despite lifestyle measures and conventional pharmacotherapy [27]. Given that MASLD is projected to become the principal driver of cirrhosis and hepatocellular carcinoma in the coming years [28], the prompt recognition of at-risk patients is of paramount importance to guide effective management and improve outcomes.

Recent advances, including antisense oligonucleotides targeting APOC3 (volanesorsen, olezarsen), ANGPTL3 inhibitors, and GLP-1 receptor agonists, have reshaped the therapeutic landscape.

Recent therapeutic advances, including antisense oligonucleotides targeting *APOC3* (volanesorsen, olezarsen), inhibitors of ANGPTL3 [29,30], and metabolic drugs such as GLP-1 [31,32,33] receptor agonists have opened new perspectives for the precision management of SHTG. At the best of our knowledge, no systematic synthesis has comprehensively integrated the genetic determinants, organ-specific complications, and therapeutic implications of SHTG.

This study addresses a critical knowledge gap, representing the first systematic attempt to integrate genotypic and phenotypic characterization with the evaluation of innovative therapeutic approaches, thereby providing a comprehensive perspective on disease mechanisms and potential treatment strategies. Building upon this rationale, the present systematic review aims to bridge genetic architecture, clinical phenotype, and therapeutic outcomes, emphasizing the genotype–phenotype continuum and the dual burden of pancreatitis and MASLD in SHTG.

## 2. Materials and Methods

A protocol for this review was prospectively developed, detailing the specific objectives, the criteria for study selection, the approach to assess study quality, the outcomes, and the statistical methods.

### 2.1. Search Strategy

This systematic review was conducted in accordance with the PRISMA 2020 guidelines [34]. A comprehensive search strategy was developed in consultation with a medical librarian. We systematically queried PubMed/MEDLINE, Embase, Web of Science, and the Cochrane CENTRAL database for the period of January 2005 through August 2025, covering the era of widespread use of next-generation sequencing (NGS).

The search combined controlled vocabulary and free-text terms related to “severe hypertriglyceridemia,” “familial chylomicronemia syndrome,” “APOC3,” “APOB,” “lipoprotein lipase,” “genetic testing,” “polygenic risk score,” “metabolic dysfunction-associated fatty liver disease (MASLD),” “volanesorsen,” “olezarsen,” and “GLP-1 receptor agonists.” No language restrictions were applied. Bibliographies of included papers and relevant reviews were hand-searched for additional references.

### 2.2. Eligibility Criteria

We included observational cohorts, case–control studies, cross-sectional analyses, registries, and interventional trials enrolling ≥ 1 human participants with triglycerides ≥ 500 mg/dL. Eligible studies had to report either results of genetic testing (targeted panels, whole-exome sequencing (WES), whole-genome sequencing (WGS), or polygenic scores) or clinical outcomes of therapeutic interventions in severe or refractory hypertriglyceridemia. Single case reports, animal or in vitro studies, editorials, and conference abstracts without extractable data were excluded.

### 2.3. Study Selection, Data Extraction, and Quality Assessment

Two reviewers (CDL, PC) independently screened titles, abstracts, and full texts, and discrepancies were resolved according to predefined criteria, with disagreements resolved by consensus or arbitration by a third reviewer (ILC). Data were extracted from eligible observational studies, registries, and randomized controlled trials. Extracted variables included study design, population size, baseline triglyceride levels, comorbidities (diabetes, hypertension, metabolic syndrome, smoking), hepatic steatosis or MASLD prevalence, pancreatitis, major cardiovascular events, and genetic background (monogenic vs. polygenic). Between-study heterogeneity was addressed by stratifying data according to study design (interventional vs. observational) and genetic classification (monogenic vs. polygenic) and by cross-checking for overlapping cohorts. Given the characteristics of the included studies, the evaluation of methodological quality of each study was performed with the ROBINS-I [35] for observational studies and RoB-2 for interventional trials [36].

### 2.4. Statistical Analysis

Descriptive statistics were applied to summarize the data, with continuous variables expressed as mean ± standard deviation (SD) and categorical variables as percentages. Due to the substantial heterogeneity among studies, no quantitative meta-analysis was performed.

## 3. Results

### 3.1. Study Selection and Characteristics

The literature search across PubMed, Embase, Web of Science, and CENTRAL initially identified 512 records, while an additional 38 records were retrieved through manual searches of reference lists and citation tracking, yielding a total of 550 records. After removal of duplicates, 430 unique records remained for screening. Based on titles and abstracts, 370 records were excluded as they were unrelated to severe hypertriglyceridemia, genetic determinants, or therapeutic interventions. Of these, 50 were excluded due to wrong population (n = 20), ineligible study design (case reports or series n = 16), or non-extractable outcomes (n = 14) (Supplementary Figure S1).

Ultimately, 10 studies met the inclusion criteria and were incorporated into the qualitative and quantitative synthesis, encompassing 2521 patients with severe or refractory hypertriglyceridemia (SHTG) [15,32,37,38,39,40,41,42,43,44]. Risk of bias and quality assessment of the included studies was performed and is reported in Supplemental Table S1.

### 3.2. Study Populations and Triglyceride Levels

Patient cohorts were heterogeneous, including individuals with monogenic familial chylomicronemia syndrome (FCS), multifactorial chylomicronemia (MCS), and metabolic hypertriglyceridemia associated with nonalcoholic or metabolic dysfunction-associated steatotic liver disease (MASLD).

Baseline triglyceride (TG) concentrations ranged from 500 mg/dL in metabolic trials to >3000 mg/dL in FCS cohorts, with a clear gradient of severity according to genotype (Table 1 and Table 2). Biallelic pathogenic variants in *LPL* or *APOC2* accounted for <5% of all cases but were associated with the most extreme phenotypes (mean TG > 2800 mg/dL) and the highest burden of pancreatitis (>70% with ≥1 prior episode). Carriers of APOA5, GPIHBP1, or LMF1 variants exhibited intermediate TG levels (1800–2500 mg/dL) and substantial pancreatitis risk, whereas APOB and APOC3 variant carriers had milder TG elevations (500–1500 mg/dL) but were more frequently associated with hepatic steatosis and metabolic comorbidities. Polygenic hypertriglyceridemia represented approximately 70–80% of all cases, with median TG levels 2200 mg/dL and pancreatitis prevalence of 15–20%, modulated by secondary factors such as obesity, alcohol intake, and uncontrolled diabetes. MASLD was present in >70% of polygenic cases, compared with <10% among monogenic FCS patients.

### 3.3. Therapeutic Outcomes

**Figure 1:**
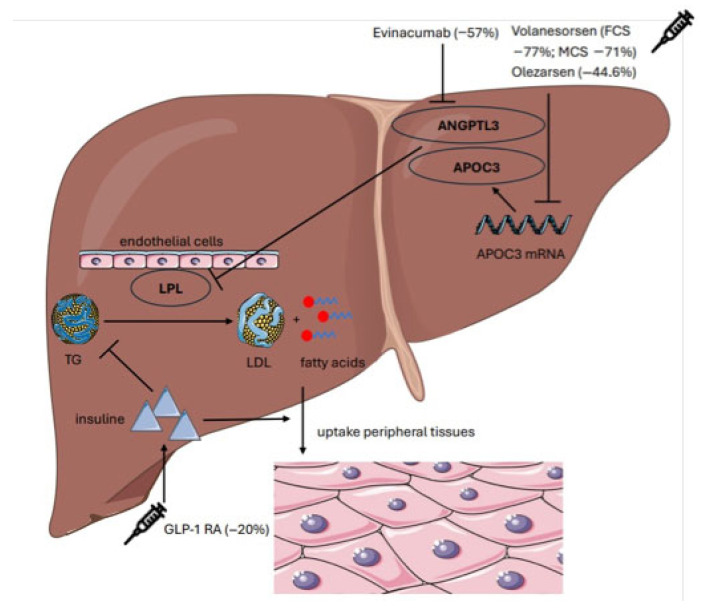
Summary of the mechanisms of action of Volanesorsen, Olezarsen, Evinacumab, and GLP-1 receptor agonists (GLP-1RA) in relation to triglyceride (TG) reduction. The percentage reduction in TG levels for each drug, relative to baseline values, is indicated in parentheses. LPL = Lipoprotein Lipase; TG = Triglycerides; LDL = Low-Density Lipoprotein; HDL = High-Density Lipoprotein; ApoC-III = Apolipoprotein C-III; ANGPTL3 = Angiopoietin-Like Protein 3; GLP-1 RA = Glucagon-Like Peptide-1 Receptor Agonist; FCS = Familial Chylomicronemia Syndrome; MCS = Multifactorial Chylomicronemia Syndrome; mRNA = messenger Ribonucleic Acid.

Emerging therapies demonstrated marked efficacy: *APOC3* antisense oligonucleotides (Volanesorsen, Olezarsen) reduced TG by ~70% with TG level targets achieved in most treated patients. ANGPTL3 inhibition achieved significant but lower TG reductions (−50–55%) with a favorable safety profile. Metabolic agents such as GLP-1 receptor agonists led to modest TG reductions (≈20–25%) but to a significant improvement in hepatic fat content (−30–35%) and resolved steatosis in up to one-third of patients (Figure 1).

### 3.4. Genotype–MASLD Relationship

**Figure 2:**
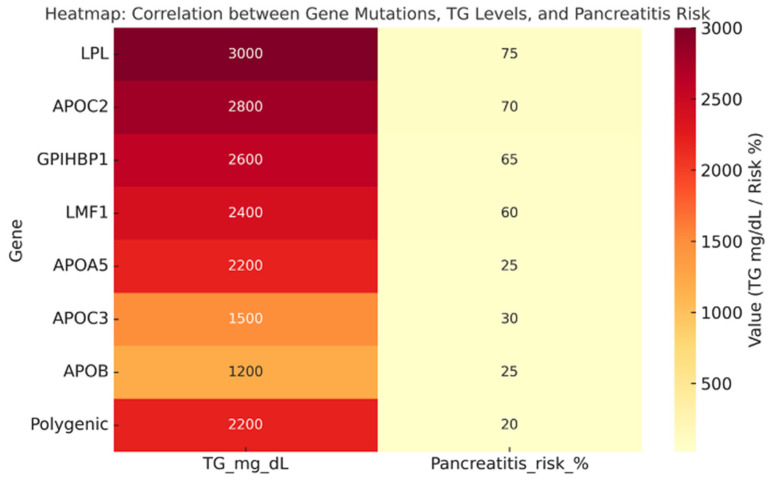
Heatmap depicting the relationship between genotype, fasting triglyceride levels, and pancreatitis risk. Each row represents a genetic determinant of hypertriglyceridemia, including rare biallelic pathogenic variants (LPL, APOC2, GPIHBP1, LMF1, APOA5), heterozygous variants (APOB, APOC3), and polygenic hypertriglyceridemia. Color intensity reflects the average triglyceride concentration (mg/dL) and reported pancreatitis frequency (%) for each genotype, with darker shades indicating more severe phenotypes. The figure highlights the severity gradient from monogenic forms (LPL/APOC2) with markedly elevated TG levels (>2800 mg/dL) and high pancreatitis burden (>70%) to polygenic and APOB/APOC3-associated forms, which display lower TG levels and reduced pancreatitis risk but are more frequently associated with metabolic comorbidities and MASLD. This visualization underscores the importance of genotype-based stratification for personalized risk assessment and therapeutic targeting in severe hypertriglyceridemia. Data were extracted from Dron et al., J Clin Lipidol 2019 [37], and Deshotels et al., Arterioscler Thromb Vasc Biol 2022 [38], and cross-validated with interventional trial cohorts (Gaudet et al., NEJM 2019 [4]; Witztum et al., Lancet 2020 [39]). LPL = Lipoprotein Lipase; APOC2 = Apolipoprotein C-II; GPIHBP1 = Glycosylphosphatidylinositol-anchored High-Density Lipoprotein Binding Protein 1; LMF1 = Lipoprotein Maturation Factor 1; APOA5 = Apolipoprotein A-V; APOC3 = Apolipoprotein C-III; APOB = Apolipoprotein B; TG: triglycerides.

**Figure 3:**
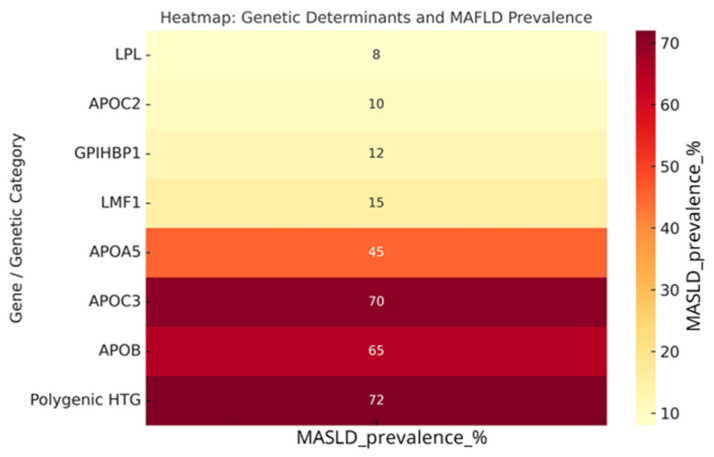
Heatmap illustrating the association between genetic determinants of severe hypertriglyceridemia (SHTG) and the prevalence of metabolic dysfunction-associated steatotic liver disease (MASLD). Each row represents a gene or genetic category (monogenic FCS variants, *APOA5/APOB/APOC3* variants, or polygenic hypertriglyceridemia). Color intensity reflects the prevalence of MAFLD, with darker shades indicating higher frequency. The figure highlights the low prevalence of MAFLD in patients with biallelic *LPL, APOC2, GPIHBP1*, or *LMF1* variants (<15%), contrasted with the high prevalence (>65%) observed in *APOC3/APOB* carriers and polygenic SHTG, supporting a “two-hit” model of hepatic overproduction and systemic TG excess. Data were extracted from Dron et al., J Clin Lipidol 2019 [37], and Deshotels et al., Arterioscler Thromb Vasc Biol 2022 [38]. LPL = Lipoprotein Lipase; APOC2 = Apolipoprotein C-II; GPIHBP1 = Glycosylphosphatidylinositol-anchored High-Density Lipoprotein Binding Protein 1; LMF1 = Lipoprotein Maturation Factor 1; APOA5 = Apolipoprotein A-V; APOC3 = Apolipoprotein C-III; APOB = Apolipoprotein B; TG: triglycerides.

A consistent finding across the included studies was the differential association between genotype and hepatic steatosis prevalence. MASLD was rare (<10%) among patients with FCS caused by biallelic loss-of-function variants in LPL, APOC2, GPIHBP1, or LMF1, likely reflecting the absence of hepatic overproduction of triglyceride-rich lipoproteins in these monogenic forms. In contrast, polygenic hypertriglyceridemia and carriers of APOC3 or APOB variants exhibited a markedly higher prevalence of MASLD (65–70%). APOC3 gain-of-function variants were associated not only with elevated plasma TG but also with increased hepatic fat content and insulin resistance, supporting the concept of a shared metabolic architecture. The heatmap analysis (Figure 2) confirmed a strong positive correlation between genotype severity and both TG levels and pancreatitis risk, emphasizing the need for genotype-driven risk stratification. A heatmap summarizing genotype–MASLD associations confirmed this gradient, showing very low prevalence of steatosis (<15%) in patients with biallelic variants in LPL, APOC2, GPIHBP1, or LMF1, and markedly higher rates (>65%) among APOC3/APOB variant carriers and polygenic cases, reinforcing the ‘two-hit’ model of hepatic overproduction and systemic TG excess (Figure 3). This gradient, from severe monogenic forms (LPL/APOC2) to milder polygenic cases, supports a precision-medicine approach where TG-lowering agents are prioritized for FCS/high-risk patients, while combined TG-lowering and metabolic therapies are particularly appropriate for MCS + MASLD phenotypes.

## 4. Discussion

This systematic review highlights key insights into the genetic architecture, clinical phenotype, and therapeutic landscape of severe and refractory hypertriglyceridemia (SHTG/rHTG). Although, the diagnostic yield of genetic testing for pathogenic variants in canonical FCS genes remains low (0.5–5%) [45], testing is clinically valuable for clarifying disease etiology, stratifying pancreatitis risk, and apprising access to novel therapies. Observational evidence described by Dron et al. confirm this phenotypic gradient showed that monogenic cases accounted for <5% of all SHTG, characterized by extreme TG elevations (>2800 mg/dL) and pancreatitis in over 70% of patients, whereas Deshotels et al. found that polygenic or multifactorial cases comprising nearly 80% of the population presented with more moderate TG elevations (~2200 mg/dL) but a strikingly higher prevalence of MAFLD (>70%) [14,37,38]. The predominance of polygenic backgrounds emphasizes the need to move beyond a dichotomous FCS–MCS model and adopt an integrated framework that combines rare monogenic variants, polygenic risk scores, and metabolic context. This genetic continuum translates into marked clinical variability, where overlapping molecular mechanisms, ranging from defective lipolysis to hepatic overproduction of triglyceride-rich lipoproteins, shape both disease severity and treatment responsiveness. Our review aims to underscore this heterogeneity, showing that most SHTG cases are polygenic, where therapeutic response is often incomplete and the residual risk of pancreatitis and MASLD-related complications remains high [46,47]. Cohort studies indicate that 40–60% of rHTG patients are carriers of imaging- or biomarker-confirmed hepatic steatosis, suggesting that steatosis and high triglyceride-rich lipoproteins levels represent two interrelated components of a shared metabolic architecture. From a pathophysiological perspective, the coexistence of genetic variants related to hypertriglyceridemia [48] and hepatic fat accumulation supports a “dual-hit” model, in which triglyceride overproduction exacerbates hepatic steatosis and insulin resistance, perpetuating the cycle of refractory hypertriglyceridemia [49]. This “dual-hit” scenario characterized by hepatic overproduction of VLDL particles and systemic hypertriglyceridemia may explain the partial refractoriness of this group to conventional TG-lowering therapy and underscores the rationale for combination strategies targeting both circulating triglycerides and hepatic lipid deposition.

Within this framework, *APOC3* and *APOB* are particularly relevant. *APOC3* gain-of-function variants are associated with elevated TG levels and pancreatitis [50], whereas loss-of-function variants confer lifelong protection. *APOB* regulates VLDL assembly and secretion, and hypomorphic variants can paradoxically worsen TG accumulation [51], further highlighting their role in disease modulation. An intriguing aspect is the so-called “*APOC3 paradox*”: gain-of-function variants not only markedly increase plasma triglycerides and pancreatitis risk but also frequently coexist with hepatic steatosis and insulin resistance, suggesting a more complex pathophysiology than impaired chylomicron clearance alone [52]. Conversely, loss-of-function variants, while protective against cardiovascular disease and pancreatitis, do not consistently result in lower hepatic fat content [50]. This dual effect underscores that *APOC3* modulation differentially affects circulating triglyceride metabolism and hepatic lipogenesis, supporting therapeutic strategies that combine *APOC3* inhibition with insulin-sensitizing or steatosis-targeting agents to disrupt the vicious cycle of hypertriglyceridemia and fatty liver. Evidence from the studies included in this review further illustrates how genetic background shapes both disease severity and therapeutic response. In patients with monogenic FCS, targeting the lipolytic pathway through *APOC3* inhibition achieved the most pronounced triglyceride reductions. The APPROACH and COMPASS trials, as well as pooled analyses, consistently demonstrated 70–80% reductions in TG levels with volanesorsen, confirming its efficacy in patients with absent or severely impaired LPL activity [4,39]. The next-generation antisense oligonucleotide olezarsen provided slightly lower efficacy (≈45% TG reduction) [53,54] with a markedly improved safety profile, while ANGPTL3 inhibition with evinacumab offered an additional therapeutic option (≈60% TG reduction) and enhanced remnant clearance even in mixed-etiology SHTG [43].

In contrast, the response in polygenic and multifactorial forms was more variable. GLP-1 receptor agonists, although less potent for triglyceride lowering (20–30%), showed substantial benefit at hepatic and metabolic endpoints, achieving 30–35% reductions in liver fat and histologic resolution of NASH in up to 59% of patients [32]. These findings highlight that while monogenic forms respond best to potent triglyceride-lowering therapies, polygenic or metabolic phenotypes require combination strategies that also address hepatic steatosis and insulin resistance.

*APOC3* and *ANGPTL3* inhibitors provide potent triglyceride reduction in monogenic and refractory forms, while metabolic agents such as GLP-1 receptor agonists target the hepatic and insulin-resistant components characteristic of polygenic or MASLD-associated hypertriglyceridemia. This integrated therapeutic framework underscores the transition from a “one-size-fits-all” approach to precision management tailored to the underlying molecular and metabolic drivers of disease [55].

Looking forward, combination strategies represent the most promising avenue. Pairing APOC3 or ANGPTL3 inhibitors with metabolic agents such as GLP-1/GIP receptor agonists may simultaneously address triglyceride burden, hepatic steatosis, pancreatitis risk, and cardiometabolic outcomes [56,57]. Genotype-guided patient selection, including *APOC3* carriers with MASLD, and integration of polygenic risk scores may further refine treatment algorithms and improve cost-effectiveness by targeting therapy to those most likely to benefit. Overall, these data define a coherent gradient reported as heatmap representation of pharmacologic responsiveness. At one end of the spectrum, low-PRS, non-monogenic patients with insulin-resistant steatosis respond best to GLP-1 receptor agonists (i.e., semaglutide, tirzepatide). Moving along the gradient, individuals with a high PRS but without major pathogenic variants represent a polygenic form of hypertriglyceridemia that responds partially to ANGPTL3 inhibition with evinacumab, reflecting preserved but inefficient lipolytic function [38,43]. The most severe zone of the heatmap, defined by coexistent PVs and high PRS, corresponds to the phenotype of multifactorial chylomicronemia, where conventional lipid-lowering agents are insufficient and APOC3 antisense therapies (volanesorsen, olezarsen) achieve maximal efficacy through direct suppression of apoC-III–mediated triglyceride accumulation [15]. Thus, increasing genetic burden parallels therapeutic specificity, highlighting the need for pathway-targeted interventions that bypass impaired lipolysis. These relationships appear consistent across ethnic groups, as demonstrated in the olezarsen trial in Japanese Americans [2], where pharmacokinetics and efficacy mirrored those observed in white cohorts. Together, these observations reinforce the concept that integrating genetic profiling into clinical trial design can help to stratify patients not only by disease severity but also by expected drug responsiveness, enabling a truly genotype-informed approach to lipid-lowering therapy.

This review has limitations. The evidence remains heterogeneous, with substantial variability in study design, sample size, and patient selection. Genetic testing yield is influenced by differences in sequencing platforms and variant classification, limiting comparability across studies. Although we included emerging data on novel therapies, most clinical trials are early phase, and long-term outcomes on pancreatitis recurrence, cardiovascular risk, and liver endpoints remain to be determined. Finally, as a narrative synthesis without formal meta-analysis, our results should be interpreted as a qualitative integration rather than definitive pooled estimates.

### Perspectives

The integration of genetic testing with detailed metabolic profiling represents the next step toward precision management of severe and refractory hypertriglyceridemia. However, the interpretation of our findings should be approached with caution, as the present synthesis is primarily qualitative and intended to highlight mechanistic and conceptual trends rather than to establish quantitative associations. As MASLD is projected to become the leading cause of cirrhosis and hepatocellular carcinoma worldwide, early identification and intervention in patients with combined hypertriglyceridemia and steatosis are crucial to alter the natural history of disease. Future research should focus on composite endpoints encompassing triglyceride lowering, prevention of acute pancreatitis, and histological or imaging-based improvement of hepatic steatosis and fibrosis. From a pharmacoeconomic standpoint, the rising burden of MASLD-related cirrhosis, hepatocellular carcinoma, and liver transplantation will impose a substantial cost on health systems, highlighting the need for cost-effective strategies. The development of APOC3 and ANGPTL3 inhibitors offers unprecedented efficacy in triglyceride reduction, but their implementation will require robust cost–utility analyses to justify reimbursement, particularly in polygenic HTG patients with MASLD, who are at the highest risk. Combining potent TG-lowering therapies with metabolic agents such as GLP-1/GIP receptor agonists may provide synergistic benefits, simultaneously reducing plasma triglycerides, hepatic fat content, and cardiometabolic risk, and ultimately decreasing downstream costs associated with advanced liver disease. Establishing prospective registries and real-world evidence programs will be key to evaluating long-term effectiveness, safety, and economic impact, enabling health systems to prioritize interventions for the patients most likely to benefit.

## 5. Conclusions

SHTG is a genetically and clinically heterogeneous disorder in which genotype dictates phenotype and therapeutic need. Monogenic FCS is characterized by extreme TG elevations and a high risk of recurrent pancreatitis, requiring potent TG-lowering interventions to prevent life-threatening events. In contrast, polygenic/MCS forms are tightly linked to MASLD, supporting a “two-hit” model where hepatic TG overproduction and systemic lipolytic impairment act synergistically. Integrating genetic testing and polygenic risk scoring into clinical practice enables precision-medicine strategies that align therapy to disease mechanism, prioritizing APOC3/ANGPTL3 inhibitors in FCS and combined TG-lowering and metabolic therapies in MCS, reducing pancreatitis recurrence, mitigating liver disease progression, and improving long-term cardiometabolic outcomes. Ultimately, a unified genomic–metabolic framework will be essential to translate genetic discoveries into actionable clinical strategies, transforming severe hypertriglyceridemia from a refractory lipid disorder into a preventable metabolic disease.

## Figures and Tables

**Table 1:** Summary of interventional and observational studies included in the systematic review. This table summarizes randomized controlled trials and meta-analyses evaluating triglyceride-lowering and hepatic outcomes in patients with severe or refractory hypertriglyceridemia, along with key observational studies describing genetic and phenotypic features. All numeric data were extracted from full-text tables or supplementary materials of the cited studies and independently verified by two reviewers for consistency.

Study	Type of Study	Intervention	Population	TG Reduction (%)	Clinical Outcomes
Calcaterra et al. (2022) [15]	Meta-analysis	Volanesorsen	66	−77%	No pancreatitis
Witzum et al. (2019) [39]	RCT	Volanesorsen,	114	−71%	QoL improvement
Bergmark et al. (2023) [40]	RCT	Olezarsen	202	−44.6%	↑ HDL + 39% ↓ ApoC-III − 65%
Rosenson et al. (2022) [43]	Meta-analysis	Evinacumab	96	−57%	LDL-C − 45% ApoCIII − 71%,
Newsome et al. (2021) [41]	RCT	Semaglutide	320	−27%	59% NASH resolution
Saddique et al. (2023) [44]	Meta-analysis	Olezarsen	202	−44%	Consistent TG lowering; improved tolerability
Liao et al. (2023) [42]	Meta-analysis	GLP-1R	859	n.r.	Liver fat reduction 30–35%.
Deshotels et al. (2022) [38]	Observational	n.a.	79	n.a.	Pancreatitis risk assessment by genotype.
Dron et al. (2019) [37]	Observational	n.a.	563	n.a.	Genetic landscape of severe HTG.
Karwatowska-Prokopczuk et al. (2024) [2]	RCT	Olezarsen	20	−73.8%	ApoC-III − 81.6% TG − 73.8%

TG = Triglycerides; QoL = Quality of Life; HDL = High-Density Lipoprotein; ApoC-III = Apolipoprotein C-III; LDL-C = Low-Density Lipoprotein Cholesterol; NASH = Non-Alcoholic Steatohepatitis; GLP-1R = Glucagon-Like Peptide-1; Receptor; HTG, hypertriglyceridemia; n.r. = not reported; n.a. = not applicable.

**Table 2:** Genetic architecture, triglyceride levels, and organ-specific phenotype in severe and refractory hypertriglyceridemia. This table summarizes the frequency of key monogenic and polygenic variants associated with severe hypertriglyceridemia (SHTG), together with corresponding triglyceride levels, prevalence of pancreatitis and metabolic dysfunction-associated fatty liver disease (MASLD), and phenotypic characteristics. Data were extracted from the full-text and supplementary materials of the included studies (Dron et al., 2019 [37]; Deshotels et al., 2022 [38]) and cross-verified for consistency.

Genotype	Prevalence	TG Levels (mg/dL)	Phenotype
*LPL* (biallelic)	1.1% of SHTG (FCS)	3000	Classic FCS, chylomicronemia, recurrent pancreatitis
*APOC2* (biallelic)	<1% (very rare)	2500–3000	FCS phenotype, poor response to fibrates/omega-3
GPIHBP1	<2%	>2500	Chylomicronemia, xanthomas, lipemia retinalis
*LMF1*	<1%	>2000	Severe HTG with variable penetrance
*APOA5*	10–15% (heterozygotes common)	1800–2500	Intermediate phenotype, often with secondary triggers
*APOC3*	Rare (<5%)	500–1500	Associated with hepatic steatosis, insulin resistance
*APOB*	Rare (<5%)	800–1500	Variable phenotype, altered VLDL distribution
Polygenic HTG (PRS ≥ 90° percentile)	≈32–47%	2200	Most common cause of SHTG, influenced by environment
Isolated heterozygotes	15–20% of Polygenic HTG patients	500–1500 mg/dL	Pancreatitis in 10–15%
Genetically uncharacterized	≈50% (no rare SNV/CNV, PRS not extreme)	2300	Likely environmental/epigenetic drivers

LPL = Lipoprotein Lipase; APOC2 = Apolipoprotein C-II; GPIHBP1 = Glycosylphosphatidylinositol-anchored High-Density Lipoprotein Binding Protein 1; LMF1 = Lipoprotein Maturation Factor 1; APOA5 = Apolipoprotein A-V; APOC3 = Apolipoprotein C-III; APOB = Apolipoprotein B; SHTG = Severe Hypertriglyceridemia; FCS = Familial Chylomicronemia Syndrome; TG = Triglycerides; HTG = Hypertriglyceridemia; VLDL = Very Low-Density Lipoprotein; PRS = Polygenic Risk Score; SNV = Single Nucleotide Variant; CNV = Copy Number Variant.

## Data Availability

No new data were created or analyzed in this study. Data sharing is not applicable to this article.

## Supplementary Materials

The following supporting information can be downloaded at: https://www.mdpi.com/article/10.3390/genes16111377/s1. Figure S1. Study selection PRISMA flow-chart. Table S1. Risk of Bias and Quality Assessment of Included Studies.
