# Supplementary material for: Genetic Assessment and Clinical Correlates in Severe Hypertriglyceridemia: A Systematic Review
Source: Genes (Basel). 2025 Nov 11;16(11):1377. doi: 10.3390/genes16111377 (PMC12652249; doi:10.3390/genes16111377)
Supplement: Supplementary file 1 [file genes-16-01377-s001.zip › genes-3917396-supplementary.pdf]

Supplementary online material

Genetic Assessment and Clinical Correlates in Severe Hypertriglyceridemia: A Systematic Review

Carmine De Luca MD[1§], Paola Ciciola PhD[1§], Guido D'Errico MD[1], Donatella Di Taranto PhD[2] Giuliana Fortunato PhD[2], Carina Gross[3], Jonathan Garn[3], Gabriella Iannuzzo MD PhD[1], Matteo Di Minno MD PhD[1*], Ilenia Calcaterra MD PhD[1*]

* These authors contributed equally to this work

§ These authors contributed equally to this work

Table of contents

**Supplemental Figure S1**	**Study selection PRISMA flow-chart**
**Supplemental Table S1**	**Risk of Bias and Quality Assessment of Included Studies**

Supplemental Figure S1: Study selection PRISMA flow-chart

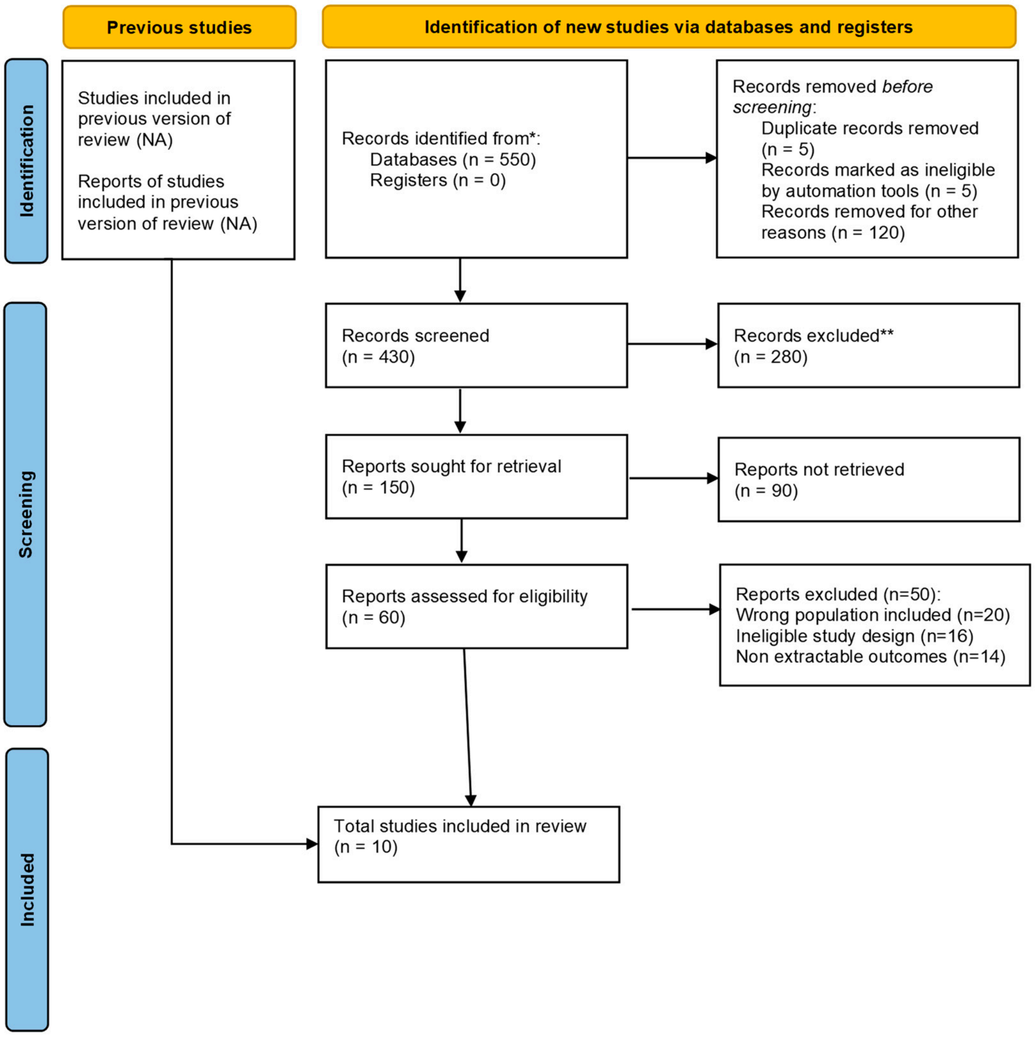

NA: not applicable

Supplemental Table S1: Risk of Bias and Quality Assessment of Included Studies

Study / Year	Design	Tool Applied	Selection Bias	Performance Bias	Detection Bias	Attrition Bias	Reporting Bias	Risk of Bias	Quality Judgment
Newsome et al. (2021)	RCT	RoB 2.0	Low	Low	Low	Low	Low	Low	Double-blind randomized design; robust endpoint adjudication
Witzum et al. (2019)	RCT	RoB 2.0	Low	Low	Low	Low	Low	Low	High internal validity; blinded outcome assessment
Bergmark et al. (2023)	RCT	RoB 2.0	Low	Low	Low	Low	Low	Low	Adequate concealment and statistical transparency
Deshotels et al. (2022)	Observational cohort	ROBINS-I	Moderate	NA	Moderate	Low	Moderate	Moderate	Large genetic cohort; no adjustment for confounders
Dron et al. (2019)	Observational cohort	ROBINS-I	Low	NA	Low	Low	Low	Low	Well-characterized cohort; minimal missing data
Rosenson et al. (2022)	RCT	RoB 2.0	Low	Low	Low	Low	Low	Low	Robust design; predefined histological endpoints
Calcaterra et al. (2022)	Meta-analysis (RCTs)	NA	NA	NA	NA	NA	NA	NA	Based on published RCTs; no additional bias introduced
Saddique et al. (2023)	Meta-analysis (RCTs)	NA	NA	NA	NA	NA	NA	NA	Based on published RCTs; no additional bias introduced
Karwatowska-Prokopczuk et.al (2024)	RCT	RoB 2.0	Low	Low	Low	Low	Low	Low	Robust design; predefined histological endpoints
Liao et. al (2023)	Meta-analysis (RCTs)	NA	NA	NA	NA	NA	NA	NA	Based on published RCTs; no additional bias introduced

Risk of bias was assessed using the Cochrane Risk of Bias (RoB 2.0) tool for randomized controlled trials and the ROBINS-I tool for observational studies. Classifications were assigned independently by two reviewers, with discrepancies resolved by consensus. 'Low', 'Moderate', and 'High' indicate the overall bias judgment. NA = not applicable.
